# Paradoxical down-regulation of p16^INK4a^ mRNA with advancing age in Acute Myeloid Leukemia

**DOI:** 10.18632/aging.100096

**Published:** 2009-10-23

**Authors:** Hendrik J.M. de Jonge, Carolien M. Woolthuis, Eveline S.J.M. de Bont, Gerwin Huls

**Affiliations:** ^1^ Division of Pediatric Oncology/Hematology, Department of Pediatrics, Beatrix Children's Hospital, University Medical Center Groningen, University of Groningen, Groningen, The Netherlands; ^2^ Department of Hematology, University Medical Center Groningen, University of Groningen, Groningen, The Netherlands

**Keywords:** p16INK4a, aging, Acute Myeloid Leukemia, senescence

## Abstract

Aging is generally considered to be the consequence of stem cell attrition caused by the activity of tumor suppressor pathways that censor potentially malignant clones by eliciting apoptosis or senescence. An important effector of aging is the cyclindependent kinase inhibitor p16^INK4a^, which is also a known suppressor of cancer. The expression of p16^INK4a^ is very low or absent in young organisms but increases with advancing age. We recently showed that, unlike healthy cells, acute myeloid leukemia (AML) derived blasts show a down-regulation of p16^INK4a^ mRNA with increasing age. Based on this observation we hypothesize that suppression of defense mechanisms which protect older cells against cellular and DNA damage might facilitate oncogenesis in older individuals.

## Aging

In human biology aging is accompanied by a diminished capacity to adequately maintain tissue homeostasis or to repair tissues after injury. When homeostatic control diminishes to a point at which tissue/organ integrity and function are no longer sufficiently maintained, physiologic decline ensues, and aging is manifested. Consistent with this, many of the pathophysiological conditions afflicting the elderly, such as anemia, sarcopenia and osteoporosis, suggest an imbalance between cell loss and renewal.

Numerous theories have been put forth to explain the decline of stem cell function with advancing age. The free-radical theory of aging proposes that reactive oxygen species (ROS), which are by-products of normal metabolism, are responsible for damage to many cellular components, including DNA [1].  It is clear, however, that in addition to ROS, a much broader range of extrinsic and intrinsic sources, such as UV irradiation, alkylating agents, telomere attrition, and DNA replication errors, can also infringe upon genomic integrity [2]. In response to this damage, tumor suppressor pathways are activated, including those mediated by the tumor suppressor proteins p16 and p53, to ensure that potentially dangerous lesions do not lead to malignancy [2,3]. So, when aging advances and damage accumulates the activity of these tumor suppressors increases and consequently has the potential to negatively modulate stem cell function through the induction of apoptosis or senescence.

## p16^INK4a ^and aging

The cyclin-dependent kinase inhibitor p16^INK4a^ has emerged as an important player in aging and age-related disease. p16^INK4a^ has the ability to bind and inhibit the cyclin-D-dependent kinases CDK4 and CDK6. These kinases are known to have oncogenic potential and phosphorylate the retinoblastoma (Rb) family of tumor suppressors. Hence, expression of p16^INK4a^ maintains Rb-family proteins in hypophosphorylated state, which promotes binding of E2F to effect a G1 cell cycle arrest [3,4]. Several studies have shown that p16^INK4a^ expression increases markedly with advancing age in a variety of tissues [5-8]. Consequently, the increased expression of p16^INK4a ^induces an age-dependent decrease in the proliferative capacity of certain tissue-specific stem- and progenitor-cells [5-8]. Senescence characterized by p16^INK4a^ upregulation as well as telomere shortening has been observed in human and mouse cardiomyocytes and may contribute to myocardial aging [9,10]. Because p16^INK4a^ expression can be upregulated by a wide variety of stresses [4,11] it may be involved in many, maybe all, forms of senescence, and has thus recently received attention as a promising biomarker [12,13].

Also in the hematopoietic system it has been shown that the induction of p16^INK4a^ correlated with the in vivo senescence of hematopoietic stem cells [14-16]. Indeed, mice lacking BMI1, a repressor of p16^INK4a^, displayed a striking loss of hematopoietic cells which correlated with increased expression of p16^INK4a^ expression and replicative failure of stem cells [16-18]. Conversely, an increase in regenerative capacity was found in the bone marrow of p16^INK4a^ deficient mice [19]. These findings reinforce the notion that the age-associated upregulation of p16^INK4a^ restricts self-renewal and unbalances tissue homeostasis. We have confirmed increased p16^INK4a^ expression in healthy human CD34+ hematopoietic cells upon aging [20].

## Aging and Acute Myeloid Leukemia

Aging not only affects normal hematopoietic development (e.g. increased incidence of anemia), but also impacts on the clinical biology of AML. In particular, the incidence of AML increases with increasing age [21,22]. Moreover, older AML patients have a markedly reduced long-term survival due to the combination of poor chemotherapeutic tolerance and inherent chemotherapy resistance compared to younger AML patients [21-25]. AML in older patients shows also a lower frequency of favorable core-binding chromosomal abnormalities and a higher incidence of complex aberrant karyotypes [26,27]. We wondered whether these differences in clinical and cellular behavior of AML in older patients were reflected by differences in gene expression profiles. Therefore, a cohort of 525 adult AML patients was studied to compare gene expression profiles of the one-third of youngest cases (median age 31 years) with the one third of oldest cases (median age 59 years) [20]. Biological processes (represented by GO-ontologies) associated with aging in AML were compared with a published list of significantly differentially expressed GO-ontologies between young and old purified murine long-term hematopoietic stem cells. This analysis revealed that in both sets the NF-κB cascade was up-regulated and that maintenance of chromatin architecture, chromatin modification and organelle organization were down-regulated [20, 28]. Subsequently, comparison of gene expression profiles of AML samples of the 175 youngest with the 175 oldest AML patients revealed that 477 probe sets were up-regulated and 492 probe sets were down-regulated with increasing age at the significance level of P <1.0x10-5.  Additionally, two in- dependent AML gene array cohorts were used for validation. The final list of validated genes which were differently expressed depending on age in three independent AML cohorts yielded a number of interesting genes, including p16^INK4a^. The level of expression of p16^INK4a^ in AML samples during aging was reciprocal to the usual trend of an increased expression at higher age; i.e. the expression of p16^INK4a^ declined significantly with increasing age. Of note, this was only noticed in the intermediate- and unfavorable-risk group and not in the favorable-risk group and the molecularly defined subset ‘NPM1 mutant without FLT3-ITD'. Multivariate analysis revealed p16^INK4a^, besides cytogenetic risk-groups, as an independent prognostic parameter for overall survival (OS) in older (and not in younger) AML patients. Lower p16^INK4a^ expression in AML samples of patients of older age predicts for reduced OS. Further studies unraveling the regulation and molecular mechanisms responsible for the down-regulation of p16^INK4a^ during aging in AML are awaited. Striking in this perspective is our observation that BMI1, a potent repressor of p16^INK4a^, was significantly inversely correlated with p16^INK4a^ in older (P = <.001, rho = -.274, n = 175) and not in younger (P = .322, rho = -.075, n = 175) AML patients.

## Is the downregulation of p16^INK4a^ a broader phenomenon?

We wondered whether the paradoxical down-regulation of p16^INK4a ^with advancing age in AML might be a more general phenomenon, i.e. is p16^INK4a^ expression also down-regulated with advancing age in other malignancies? An extensive search in the Gene Expression Omnibus (GEO) repository revealed only three publically available Affymetrix gene array datasets of malignancies (i.e. lymphoma, glioblastoma and breast cancer) which also presented the patient characteristic age (GSE4475, GSE7696 and GSE3494) [29-31]. The median age at diagnosis is given in Table 1 for all three cohorts. Interestingly, the expression of p16^INK4a^ declined significantly with advancing age in lymphoma patient samples (P = <.001, rho = -.252 n = 219, Table 1) and in glioblastoma patient samples (P = .005, rho = -.310, n= 80, Table 1). In breast cancer patient samples the continuous variables age and p16^INK4a^ did not correlate (P = .407, rho = -.053, n = 251, Table 1).

**Table 1.** Correlation between the expression of p16 ^INK4a^ with age with age at diagnosis in three malignancies. The relation between the expression of p16^INK4a^ mRNA level and age at diagnosis in three malignancies (publicly available micro arrays, i.e. GSE4475, GSE7696 and GSE3494)[29-31]. Spearman rank correlation coefficients between the continuous variables age and the averaged p16^INK4a^ probe sets (n=3) were calculated. The characteristic age is given as median (range).

**Cancer type**	**age**	**n**	***P***	**Rho**
lymphoma	61 (2-61)	219	**<.001**	-.252
glioblastoma	52 (26-70)	80	**.005**	-.310
breast cancer	64 (28-93)	251	.407	-.053

## Conclusive remarks

**Figure 1.**
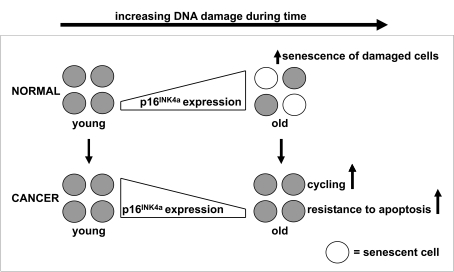
p16 ^INK4a^ expression during aging of healthy and malignant cells. The expression of p16^INK4a^ mRNA increases with advancing age to ensure that potentially dangerous lesions, due to accumulated DNA damage, do not lead to malignancy. The increased expression of p16^INK4a^ mRNA has the potential to negatively modulate stem cell function through the induction of apoptosis or senescence. Our data illustrate the importance of this p16^INK4a^ dependent mechanism, since samples of older cancer patients have a lower instead of higher expression of p16^INK4a^ mRNA compared to samples of younger cancer patients. So, we hypothesize that suppression of defense mechanisms which protect older stem cells against accumulated cellular and DNA damage facilitates the development of cancer in older individuals.

The cyclin-dependent kinase inhibitor p16^INK4a^, which has emerged as an important effector of aging and a potent tumor suppressor, was shown to be down-regulated in older AML samples [20]. However, this is not unique for AML since the paradoxical down-regulation with advancing age is also observed in samples from lymphoma and glioblastoma patients. These observations provide an interesting new link between aging and cancer. We hypothesize that suppression of defense mechanisms which protect older cells against cellular and DNA damage might facilitate oncogenesis in cancer cells from older individuals (Figure 1). Accordingly, the interaction between aging and cancer warrants further studies, to determine whether age-associated protection mechanisms play a specific role in cancer of older patients and to determine whether modulation of these age-associated protection mechanisms can be exploited in the treatment of older cancer patients.
